# The Effects of Hormone Diets with Different 17β-Estradiol Levels on Growth and Feminization in Long-Whiskered Catfish (*Mystus gulio*) Larvae Using Conventional and Microencapsulated Feed

**DOI:** 10.3390/ani16020268

**Published:** 2026-01-15

**Authors:** Sahabhop Dokkaew, Kritchavat Songdum, Noratat Prachom, Wiwiththanon Boonyung, Suwaree Kitikiew, Khwankhao Khamphet, Preecha Waicharoen, Uthairat Na-Nakorn, Natthapong Paankhao, Anurak Uchuwittayakul, Phunsin Kantha

**Affiliations:** 1 Department of Aquaculture, Faculty of Fisheries, Kasetsart University, Bangkok 10900, Thailand; ffisspd@gmail.com (S.D.);; 2 Center of Fish Feed Technology Development, Faculty of Fisheries, Kasetsart University, Bangkok 10900, Thailand; 3 Nongkhai Inland Aquaculture Research and Development Center, Department of Fisheries, Nongkhai 43130, Thailand; 4 Department of Animal Production Technology and Fisheries, School of Agricultural Technology, King Mongkut’s Institute of Technology Ladkrabang, Bangkok 10520, Thailand; 5 Kamphaeng Saen Fisheries Research Station, Faculty of Fisheries, Kasetsart University, Kamphaeng Saen Campus, Kamphaeng Saen, Nakhon Pathom 73140, Thailand; 6 Center of Excellence in Aquatic Animal Health Management, Faculty of Fisheries, Kasetsart University, Bangkok 10900, Thailand

**Keywords:** *Mystus gulio*, feminization, 17β-estradiol, microencapsulated feed, sex reversal, estrogen-related genes, aquaculture sustainability, growth performance

## Abstract

Feminization is an important biotechnological approach in aquaculture for species in which females exhibit superior growth and higher market value. The long-whiskered catfish (*Mystus gulio*), a euryhaline species cultivated in both monoculture and co-culture systems, contributes to sustainable aquaculture by grazing on uneaten feed and maintaining pond cleanliness. This study evaluated the effects of dietary 17β-estradiol (E2) at 0, 10, 30, and 60 mg/kg, incorporated into conventional and microencapsulated feeds, on the feminization and early growth of *M. gulio* larvae. Treatments were administered during the weaning stage for 14 and 21 days under controlled rearing conditions. Results showed that larvae fed microencapsulated feed containing 60 mg/kg E2 achieved the highest specific growth rate (26.91 ± 1.92%/day), feed efficiency (164.76 ± 33.23%), and feminization success (99.73 ± 0.04%). Hormonal assays confirmed elevated estradiol and reduced testosterone levels, consistent with ovarian development observed in histological sections. Gene expression analysis further supported these findings through the significant upregulation of *cyp19a*, *erb1*, and *erb2* mRNA levels. Overall, this study demonstrates that microencapsulated hormone feeding is an effective and environmentally responsible strategy for achieving monosex female populations in *M. gulio*, enhancing productivity, reproductive performance, and sustainability in aquaculture systems.

## Simple Summary

The long-whiskered catfish (*Mystus gulio*) is a valuable aquaculture species because female fish grow faster and provide higher economic returns than males. This species is commonly cultured alone or together with other aquatic animals such as seabass and shrimp, where it helps maintain cleaner pond conditions by consuming leftover feed on the bottom. It can adapt well to different salinity levels, from freshwater to seawater, making it an excellent candidate for sustainable aquaculture. In this study, young catfish were fed diets containing different levels of the natural female hormone 17 β-estradiol, provided either in regular feed or in specially designed microencapsulated feed that allows for slow hormone release. The results showed that microencapsulated diets with the highest hormone level produced faster growth, better feed use, and an almost completely female population. Microscopic and genetic analyses confirmed the successful development of ovaries in these fish. These findings demonstrate an effective and environmentally responsible technique for producing all-female catfish, improving productivity, reproductive potential, and long-term sustainability in aquaculture operations.

## 1. Introduction

Monosex culture, particularly female-only production, is a widely used strategy in aquaculture to enhance productivity and profitability. In many fish species, females exhibit faster growth rates, superior flesh quality, and higher market value compared to males [1,2]. As a result, producing all-female populations has become a key goal in aquaculture operations. One of the most effective techniques for producing monosex offsprings is hormonal treatment, with 17β-estradiol (E2) being a common technique for inducing feminization [3,4,5]. The success of feminization also depends significantly on the delivery system used for the hormone, with microencapsulation technology emerging as a promising method for controlled hormone release.

Microencapsulated feeds offer several advantages over conventional hormone-supplemented diets. The improved success of feminization depends on several factors, including the hormone dosage, the duration of exposure, and the composition of the feed [6]. Controlled release mechanisms enhance hormone absorption and minimize loss, thereby mitigating the environmental impact of leaching. Nevertheless, the efficacy of sex reversal is contingent upon the delivery system. Conventional fishmeal-based diets are prone to heterogeneous hormone distribution and rapid aqueous leaching, which compromise feminization efficacy and pose environmental risks [7,8]. While microencapsulation has demonstrated superior efficacy, its higher cost and production complexity may limit its use in smaller hatcheries [9]. The application of E2 in aquaculture for sex reversal presents both challenges and opportunities, particularly in scaleless species like *M. gulio*.

This experiment is especially relevant for *M. gulio*, commonly known as the long-whiskered catfish, a species widely cultured in Thailand. This euryhaline fish can thrive across a wide range of salinities—from freshwater to brackish and marine environments [10]. *M. gulio* is often cultured as a secondary species alongside high-value aquaculture organisms such as Asian seabass (*Lates calcarifer*), tiger shrimp (*Penaeus monodon*), freshwater giant prawn (*Macrobrachium rosenbergii*) [11], and tilapia (*Oreochromis niloticus*) [12]. In such co-culture systems, *M. gulio* contributes to system sustainability by cleaning benthic areas and grazing on leftover feed and organic matter, thereby improving water quality and overall productivity [13]. Excessive hormone exposure can lead to gonadal malformation or sterility [14], highlighting the need for a precise delivery method.

To address these challenges, researchers have turned to microencapsulation technology as a more effective delivery system. Microencapsulation helps stabilize bioactive compounds like E2, reduce leaching, and ensure gradual hormone release within the digestive system [15]. This technique not only improves hormone absorption but also increases dosing accuracy and feed palatability. Compared to traditional fishmeal-based hormone feeds, microencapsulated diets may offer higher efficiency and consistency in feminization outcomes. Despite its advantages, microencapsulation technology can be more costly and technically demanding, limiting its use in small-scale hatcheries. Traditional hormone-supplemented feeds are more affordable but often suffer from uneven hormone distribution and rapid degradation in water [16]. While microencapsulated feeds have demonstrated improved hormone delivery in other species, the optimal E2 dose and feed type for *M. gulio* have never been systematically studied.

This study aims to compare the effectiveness of microencapsulated E2 diets with conventional fishmeal-based hormone diets to evaluate their impact on growth, survival, and feminization rates in *M. gulio*, while minimizing negative physiological or environmental impacts. Comparative feeding trials were conducted using both microencapsulated and conventional hormone-supplemented feeds over two treatment durations (14 and 21 days). In addition to phenotypic assessments, histological analysis and hormonal profiling were undertaken to confirm sex differentiation outcomes. The presence of ovarian structures in histological sections, alongside elevated estradiol and suppressed testosterone levels measured via ELISA, provides evidence of successful feminization. Molecular analysis of key genes such as estrogen receptor β1 (*erb1*), estrogen receptor β2 (*erb2*), and aromatase (*cyp19a*) further supports the interpretation of physiological responses to hormone exposure.

## 2. Materials and Methods

### 2.1. Preparation of Hormonal Complex-Microencapsulated Feed

The formulation of a specialized hormonal feed for *M. gulio* larvae began with a proximate chemical analysis of fertilized eggs and 2-day-old fry to establish the species’ nutritional baseline [7]. Analyses included moisture, crude protein, crude fat, amino acid composition, and fatty acid profiles, following standard methods of the Association of Official Analytical Chemists [17]. The amino acid requirement was estimated using the amino acid to essential amino acid ratio (A/E ratio), with arginine used as a reference amino acid for comparison [18].

Feed production was carried out using a twin-screw extruder (Jinan Saixin Machinery Co., Ltd., Jinan, China) fitted with a double-layer steam conditioner, enabling precise control of thermal and mechanical processing conditions to preserve hormone integrity [19]. The extruder produced microparticles with a target diameter of 150–300 µm, suitable for ingestion by early-stage larvae. During the formulation, E2 was dissolved in ethanol and incorporated at four concentrations: 0, 10, 30, and 60 mg/kg of feed. The mixture was homogenized prior to extrusion to ensure uniform hormone distribution. After extrusion, microparticles were coated with an oil-in-water emulsion containing egg yolk and fish oil under mild vacuum and agitation to enhance palatability and minimize hormone leaching. The coated feed was sieved through a 60-mesh stainless steel screen to ensure ≥90% particle size uniformity and stored in airtight containers at ≤4 °C under moisture-free conditions until use.

### 2.2. Experimental Design and Fish Rearing

The feeding trial for *M. gulio* larvae was structured in a 2 × 2 × 4 factorial design to examine the effects of feed type, hormone dosage, and feeding periods. Two types of feed were prepared: a conventional fishmeal-based feed or normal feed (N) and a hormonal complex-microencapsulated feed (E). Four concentrations of E2 (E2) were incorporated into diets at 0, 10, 30, and 60 mg/kg. Each hormone concentration was tested under two feeding durations, 14 and 21 days, for both feed types. This experimental arrangement produced sixteen distinct treatment combinations, allowing for the evaluation of the independent and interactive influences of hormone concentration, feed formulation, and exposure duration on the growth performance, feed utilization, and feminization success of *M. gulio* larvae. All experimental procedures were approved by the Animal Care and Use for Scientific Research Committee, Kasetsart University (approved no. ACKU68-FIS-018).

Weaning-stage larvae (2 days post-hatching (dph)) were randomly distributed into 500 L cylindrical fiberglass tanks at a stocking density of 2 fish per liter (approximately 1000 fish per tank). Larvae underwent a 24 h acclimation period under controlled water conditions before the start of the treatment. All tanks were part of a closed recirculating aquaculture system (RAS) equipped with mechanical and biological filtration to ensure stable water quality throughout the trial.

### 2.3. Feeding Trial and Water Quality Monitoring

Feeding was performed manually five times daily at fixed intervals (08:00, 10:00, 12:00, 14:00, and 16:00 h), allowing for accurate monitoring of feed intake and larval behavior. Feed quantities were adjusted to achieve apparent satiation, ensuring that all groups received equivalent feeding levels. Water quality parameters, including temperature (°C), pH, and dissolved oxygen (DO, mg/L), were monitored twice daily at 06:00 and 15:00 hrs using a multi-parameter water quality meter (YSI 550-A, YSI Inc., Yellow Springs, OH, USA). Parameters were maintained within optimal ranges for *M. gulio* larviculture throughout the experiment (temperature: 28.5 ± 0.5 °C; DO: 6.0 ± 0.3 mg/L; pH: 7.7 ± 0.3). The photoperiod was maintained at 12 L:12 D (12 h light; 12 h dark) using overhead fluorescent lighting. Tanks were cleaned daily via siphoning of waste and uneaten feed to minimize ammonia buildup and ensure hygienic rearing conditions.

### 2.4. Sample Collection and Growth Performance Analysis

At the end of each treatment period (14 or 21 days), samples of *M. gulio* larvae were collected to assess growth performance, sex differentiation, and molecular and physiological responses related to feminization and development. This sampling approach enabled comprehensive evaluation of both the short-term and cumulative effects of E2 administration through different feed formulations and durations under controlled rearing conditions. Prior to sampling for hormone analysis and gene expression, larvae were starved for 24 h to ensure complete evacuation of the gastrointestinal tract. Fish were euthanized using buffered MS-222 (Tricaine methane sulfonate, cat. no. GE5936; Glentham Life Sciences Ltd., Corsham, UK) 100 mg/L, and whole-body samples were collected for downstream analyses. When individual sample weight was less than 1 g, larvae were pooled by tank prior to measurement to ensure accuracy and minimize weighing errors. The following growth performance parameters were assessed [7,20]:

- Survival rate (%): monitored daily in each tank by counting live individuals;
- Body weight (g): measured using a precision balance (±0.001 g);
- Weight gain (g) = Mean final weight (g) − Mean initial weight (g);
- Specific growth rate (SGR %/day) = (ln final weight (g) − ln initial weight (g)) × 100/experimental period in days;
- Feed conversion efficiency (FE) = (Body weight gain (g)/Total dry weight of feed (g)) × 100.

### 2.5. Sex Ratio and Gonadal Development

Gonadal tissue samples for feminization efficiency were collected from fish at 14- and 21 days post-treatment. Due to the small size of the gonads at these stages, whole-body larvae were euthanized and cross-sectioned, including all abdominal viscera (gonads, liver, and surrounding tissues) for histological sex identification. A total of 1 g of pooled tissue from each treatment group (*n* = 3 tanks/treatment) was collected. The samples were fixed in Davidson’s fixative for 24–48 h and processed using standard histological techniques [21]. After paraffin embedding, the tissues were sectioned at 5–7 µm thickness and stained with hematoxylin and eosin (H&E; Sigma-Aldrich, St. Louis, MO, USA). Gonadal structures were then differentiated under light microscopy using an Olympus CX23RTFS2 microscope (Olympus Corporation, Tokyo, Japan, No. 4D81772).

The sex of each fish was determined based on the morphological characteristics of gonadal tissues. At least 30 fish per treatment group were examined [7]. The sex ratio was calculated as the percentage of phenotypic females in each group using the following formula:

$$Sex\;Ratio\;(Female)=\left(\frac{Number\;of\;female\;fish}{Total\;number\;of\;sexed\;fish}\right)\times 100$$

### 2.6. Hormone Biochemical Parameters

Hormone concentrations in *M. gulio* larvae were assessed to confirm the effects of E2 on feminization. Testosterone (T) and estradiol (E2) levels were quantified using commercially available enzyme-linked immunosorbent assay (ELISA) kits (Cayman Chemical, Ann Arbor, MI, USA). The Cayman Testosterone ELISA Kit (no. 582701) was used to measure testosterone concentrations in whole-body pooled samples, while the Cayman Estradiol ELISA Kit (no. 501890) was used to quantify estradiol levels.

Whole-body samples (1 g pooled from larvae per treatment group) were collected at the end of the feeding trial. The samples were homogenized and extracted in ethyl acetate, followed by drying and reconstitution for analysis according to the manufacturer’s instructions. Hormonal quantification was performed using the provided protocols. The samples were shaken on an Orbital Shaker/Platform Shaker Model: 3016 (GFL Gesellschaft für Labortechnik mbH, Burgwedel, Germany), and absorbance was measured at the appropriate wavelengths for each hormone using the iMark™ Microplate Reader Serial No. 10554 (Bio-Rad Laboratories, Hercules, CA, USA). All samples were analyzed in duplicate, and the average of the two readings was used for statistical analysis.

### 2.7. Gene Expression

Gene expression analysis was conducted on head tissues, including the hypothalamus–pituitary region, to explore the molecular mechanisms underlying feminization in *M. gulio*. Total RNA was extracted using the EasySpin Total RNA Extraction Kit (Intron, Seongnam, Republic of Korea) following the manufacturer’s instructions. RNA concentration and quality were assessed using spectrophotometry (A260/A280) with a NanoDrop™ 2000c Spectrophotometer (Thermo Fisher Scientific, Wilmington, DE, USA) and confirmed through agarose gel electrophoresis using a Bio-Rad PowerPac Basic (Bio-Rad Laboratories, Hercules, CA, USA). Primers targeting *erb1*, *erb2*, and *cyp19a* (Table 1) were obtained from Integrated DNA Technologies (Singapore).

First-strand cDNA was synthesized using easy-spin™ and Maxime™ RT Premix (Oligo(dT) 15 primer) kits (Thermo Fisher Scientific, Wilmington, DE, USA) following the manufacturer’s instructions. The reaction was carried out using a SensoQuest LabCycler PCR-Thermocycler (SensoQuest GmbH, Göttingen, Germany). A total of 1 μg of total RNA was denatured at 95 °C for 5 min. Reverse transcription was then performed at 57 °C for 60 min, followed by an enzyme inactivation step at 72 °C for 5 min. The synthesized cDNA was stored at −80 °C for subsequent gene expression analysis.

The RT–qPCR reaction for all target genes was performed using first-strand cDNA as the template. Reactions were prepared in a total volume of 10 µL, consisting of 5 µL SYBR™ Green qPCR Ultra Mix, 1 µL of each forward (1.0 µM) and reverse (1.0 µM) primer, 1 µL sterile distilled water, and 2 µL first-strand cDNA template. Reactions were conducted in a 96-well plate using an AriaMx™ G8830A Real-Time PCR System (Agilent Technologies, Santa Clara, CA, USA) with the following thermal cycling conditions: initial denaturation at 95 °C for 5 min, followed by 30 amplification cycles of denaturation at 95 °C for 30 s, annealing at 55 °C for 30 s, and extension at 72 °C for 30 s. Primer specificity was confirmed by melt-curve analysis, and amplification efficiency was calculated using standard curves. Gene expression levels were normalized against a stable β-actin housekeeping gene and analyzed using the 2^−ΔΔCt^ method [22].

### 2.8. Statistical Analysis

Data were analyzed using Graphpad prism 10 (GraphPad Software, Boston, MA, USA), with two-way ANOVA and three-way ANOVA followed by Tukey’s HSD test at a 95% confidence level to assess differences in feminization rates, growth performance, and gene expression of hormonal concentrations. Shapiro–Wilk and Levene’s tests were used to check normality and homogeneity of variance, respectively. If assumptions were violated, data transformations or a Kruskal–Wallis test were applied. Results were reported as mean ± SD, with statistical significance set at *p* < 0.05.

## 3. Results

### 3.1. Nutritional Evaluation and Amino Acid Profiling of Hormonal Complex-Microencapsulated Feed for Mystus gulio Weaning Larvae

Proximate analysis of *M. gulio* fertilized eggs and 2-day-old larvae revealed a high protein and essential amino acid requirement for early development (Table 2). The proximate composition and amino acid profiles of *M. gulio* fry, fertilized eggs, and the experimental weaning feed. Fry samples exhibited the highest total crude protein content at 65.83%, followed by fertilized eggs (57.23%) and feed (39.38%). Total crude fat was highest in fertilized eggs (5.34%) and lowest in the fry (1.66%). Fertilized eggs also contained the highest levels of total essential amino acids (35.93 g/100 g) and total non-essential amino acids (29.38 g/100 g). The experimental microencapsulated feed formulation successfully replicated this profile. Amino acid profiling confirmed that the formulated diet met the theoretical requirements derived from the A/E ratio of the natural reference samples, maintaining consistency in protein quality across all treatment groups. The hormonal complex-microencapsulated feed (E feed) matched the ingestive mount-opening of *M. gulio* larvae at 3 dph. All feed formulations, including experimental diets, were subjected to proximate composition analysis to determine moisture, crude protein, crude fat, crude fiber, and ash content, as well as detailed amino acid profiling. This ensured that the experimental feed met the specific nutritional needs of *M. gulio* larvae and maintained consistency across treatments (Table 2).

In addition to nutritional profiling, the physical characteristics of the microencapsulated feed were evaluated to ensure ingestibility. Microscopic analysis revealed a uniform spherical morphology (Figure S1). This particle size distribution aligns with the mouth gape size of *M. gulio* larvae at 3 dph, facilitating efficient capture and ingestion.

### 3.2. Growth Performance, Feed Efficiency, and Sex Differentiation Rates

**Figure 1:**
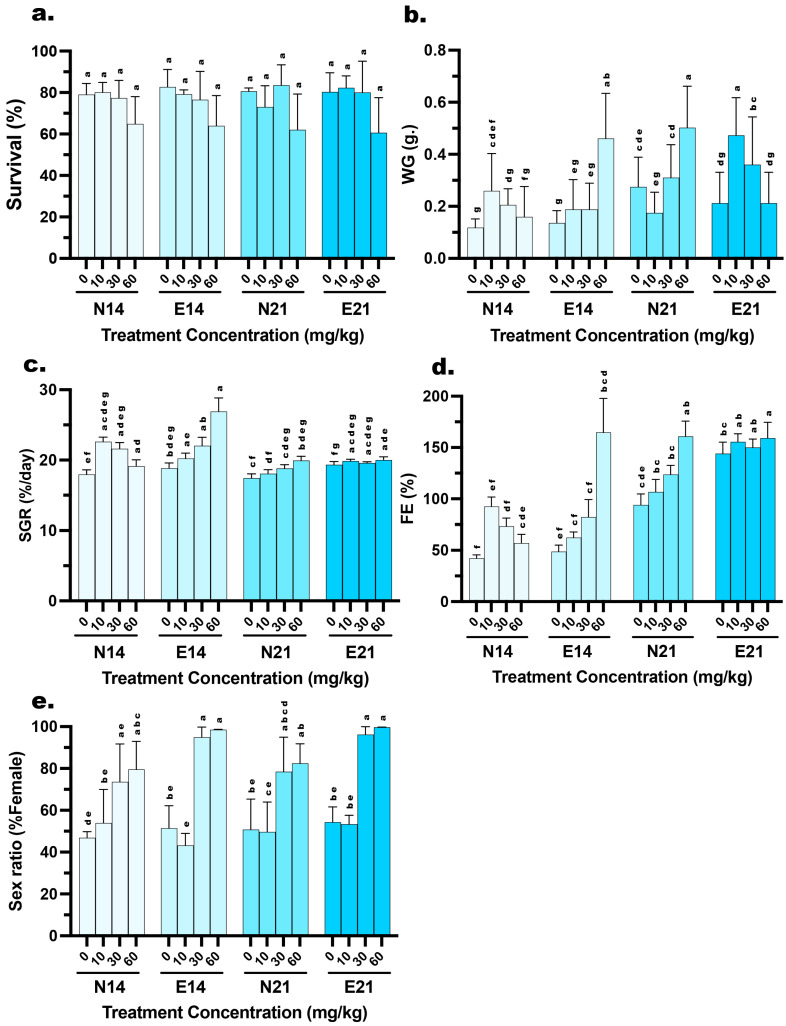
Effects of dietary 17β-estradiol (E2) concentration, delivery method, and treatment duration on the growth performance, survival, and feminization of *Mystus gulio* larvae. Larvae were administered E2 via conventional (N) or microencapsulated (E) feeds at concentrations of 0, 10, 30, and 60 mg/kg for periods of 14 days (N14, E14) or 21 days (N21, E21). The panels illustrate (**a**) survival rate (%), (**b**) weight gain (WG, g.), (**c**) specific growth rate (SGR, %/day), (**d**) feed efficiency (FE, %), and (**e**) sex ratio (% Female). Data are presented as mean values ± standard deviation (SD). Different lowercase letters indicate statistically significant differences between groups (*p* < 0.05).

Survival rates varied across experimental treatments, ranging from 60% to 81.57% (Figure 1). A three-way ANOVA evaluated the effects of Feed Type (FT), Hormone Dose (HD), and Time Period (TP) on larval survival. The analysis revealed a significant main effect for Hormone Dose (HD) (F (3, 32) = 7.084, *p* < 0.001), indicating that survival rates were dependent on the specific estradiol concentration administered. In contrast, no significant main effects were observed for FT (F (1, 32) = 0.038, *p* = 0.958) or TP (F (1, 32) = 0.003, *p* = 0.846). Furthermore, there were no significant interaction effects between factors, including HD×FT (F (3, 32) = 0.313, *p* = 0.816), HD×FT (F (3, 32) = 0.202, *p* = 0.894), TP×FT (F (1, 32) = 0.015, *p* = 0.904), or the three-way interaction HD×TP×FT (F (3, 32) = 0.258, *p* = 0.855) (Table 3).

Post hoc analysis revealed distinct weight gain (WG). At 14 days, the E feed containing 60 mg/kg E2 resulted in the highest weight gain (0.46 ± 0.17 g), which was significantly higher (*p* < 0.05) than both the lower hormone doses in the same feed group and the N feed at the same concentration (0.16 ± 0.12 g). However, by 21 days, this trend shifted. The N feed group at 60 mg/kg exhibited a surge in growth, reaching 0.50 ± 0.16 g, significantly higher (*p* < 0.05) than the E feed group, which showed reduced weight gain (0.21 ± 0.12 g) in the 21 days. Meanwhile, the 10 mg/kg E feed group showed a significant increase in WG from 14 days (0.19 ± 0.11 g) to 21 days (0.47 ± 0.14 g) (Figure 1). WG was significantly influenced by all experimental factors and their interactions. A three-way ANOVA revealed significant main effects for HD (F (3, 464) = 29.94, *p* < 0.001), FT (F (1, 464) = 6.482, *p* = 0.001), and TP (F (1, 464) = 81.56, *p* = 0.011). Furthermore, significant two-way interactions were observed for HD×FT (F (3, 464) = 3.07, *p* = 0.028), HD×TP (F (3, 464) = 7.046, *p* < 0. 001), and FT×TP (F (1, 464) = 7.027, *p* = 0.008). Most notably, a highly significant three-way interaction was found for HD×FT×TP (F (3, 464) = 81.41, *p* < 0.001) (Table 3).

Based on post hoc analysis of Specific Growth Rate (SGR), the E diet demonstrated superior performance at 14 days when the E feed group receiving 60 mg/kg E2 exhibited the highest SGR recorded 26.91 ± 1.92%, a value significantly higher (*p* < 0.05) than the N feed group at the same dosage (19.13 ± 0.91%). However, the SGR for this high-dose E feed group stabilized by 21 days (20.01 ± 0.45%), making it comparable to the N feed group (19.95 ± 0.59%). Notably, while the N feed groups at 10 and 30 mg/kg experienced a significant decline in SGR from 14 to 21 days (e.g., the 30 mg/kg group dropped from 21.61% to 18.81%), the E feed groups at corresponding doses maintained stable growth rates (e.g., the 30 mg/kg group remained consistent at 19.60%) (Figure 1). SGR exhibited significant variation across the different experimental conditions. A three-way ANOVA demonstrated significant main effects for HD (F (3, 32) = 30.84, *p* < 0.001), FT (F (1, 32) = 36.23, *p* < 0.001), and TP (F (1, 32) = 76.26, *p* < 0.001). The dependency of these factors on each other was confirmed by a significant three-way interaction HD×FT×TP: F (3, 32) = 29.61, *p* < 0.001) (Table 3).

Feed efficiency (FE) revealed a general trend of significant improvement across most treatments from 14 to 21 days. Nevertheless, the E feed demonstrated superior efficiency, particularly in specific treatment. At 14 days, the 60 mg/kg group achieved an exceptionally high FE of 164.76 ± 33.23%, which was significantly greater (*p* < 0.05) than the N feed at the same dose (57.02 ± 8.58%). While the FE of the high-dose groups converged by 21 days (159.28% vs. 160.87%), the E feed maintained a significant advantage in the lower dose ranges. Specifically, at the 21-day mark, the E feed groups at 0, 10, and 30 mg/kg exhibited significantly higher FE (ranging from 144.05% to 155.56%) compared to their N feed counterparts (ranging from 94.13% to 123.73%) (Figure 1). FE was strongly influenced by the experimental conditions, showing highly significant variations across all factors. A three-way ANOVA indicated significant main effects for HD (F (3, 32) = 33.34, *p* < 0.001), FT (F (1, 32) = 51.34, *p* < 0.001), and, most notably, TP (F (1, 32) = 242.6, *p* < 0.001). The complexity of these effects was confirmed by a significant three-way interaction (HD×FT×TP: F (3, 32) = 29.41, *p* < 0.001) (Table 3).

Analysis of sex ratios in the control (0 mg/kg) and low-dose (10 mg/kg) groups indicated approximate parity (ranging from 43.20 to 54.35% female), with no significant deviations from the expected natural ratio (1:1). Conversely, a sharp increase in feminization was observed at the 30 mg/kg and 60 mg/kg dosages (Figure 1). A three-way ANOVA revealed a highly significant main effect for HD (F (3, 32) = 50.16, *p* < 0.001), confirming that feminization is dose-dependent. Additionally, FT showed a significant main effect (F (1, 32) = 9.673, *p* = 0.004), indicating that microencapsulation improved overall feminization success compared to N feed. A significant interaction between HD×TP (F (3, 32) = 3.313, *p* = 0.032) further suggests that the efficacy of the feed type varies with the dosage used. Interestingly, despite the numerical increase in female percentages from 14 to 21 days in the high-dose groups, the main effect of TP was not statistically significant (F (1, 32) = 0.882, *p* = 0.355) (Table 3).

**Figure 2:**
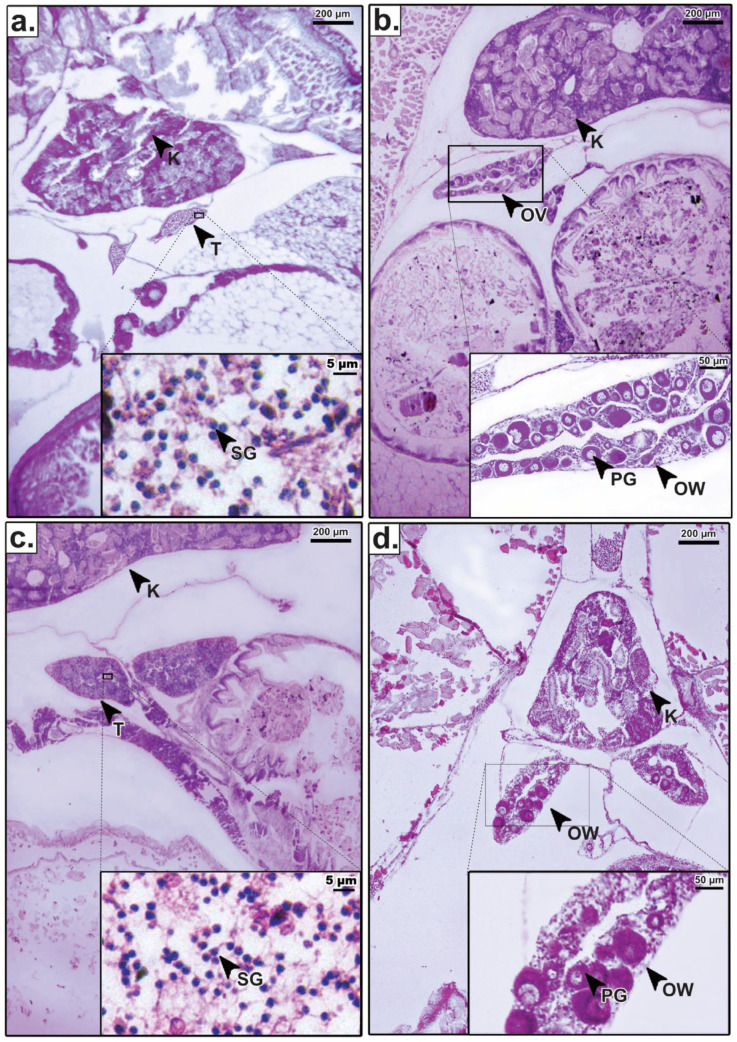
Histological sections of the gonads in *Mystus gulio* at 21 days post-hatching (dph): (**a**) testis (T) from the hormone-treated group, showing clusters of spermatogonia (SG) adjacent to the kidney (K); (**b**) ovary (OV) from the hormone-treated group, exhibiting primary growth oocytes (PG) and a well-defined ovarian wall (OW); (**c**) T from the untreated control group; (**d**) OV from the untreated control group. Scale bars: 200 µm (main images) and 50 µm (insets).

Histological analysis of *M. gulio* larvae at 21 dph was performed to confirm phenotypic sex differentiation and assess the morphological integrity of the gonads following hormone treatment (Figure 2). To validate the success of the feminization process, gonadal structures from hormone-treated individuals were compared directly against untreated controls. In the untreated control group, males exhibited typical testicular differentiation characterized by clusters of spermatogonia adjacent to the kidney (Figure 2c), while females displayed clear ovarian differentiation with primary growth oocytes enclosed within a defined ovarian wall (Figure 2d).

Crucially, the histological structure of the sex-reversed females in the hormone-treated groups (Figure 2b) was morphologically indistinguishable from that of the natural females in the control group (Figure 2d). Both exhibited healthy primary oocytes and similar tissue organization, confirming that the dietary E2 treatment induced functional ovarian differentiation without causing histological abnormalities. Similarly, the testes of males remaining in the treated groups (Figure 2a) showed normal spermatogonia clustering comparable to control males (Figure 2c).

### 3.3. Hormonal Response to Estradiol Treatments

**Figure 3:**
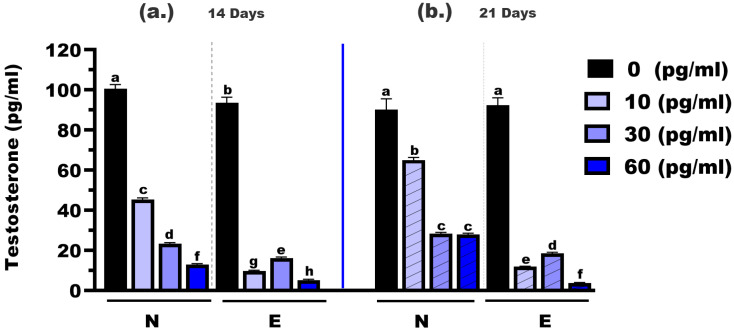
Testosterone concentrations in *Mystus gulio* larvae. Larvae were fed diets containing different 17β-estradiol levels (E2), delivered through two distinct feed types, conventional fishmeal-based feed (N) and hormonal complex-microencapsulated feed (E), over 14-day (**a**) and 21-day (**b**) treatment periods. Data are presented as mean ± standard error (SE). Concentrations are expressed in pg/mL. Data were analyzed using a one-way ANOVA, followed by Tukey’s multiple comparisons test. Different lowercase letters indicate statistically significant differences between groups (*p* < 0.05).

The testosterone concentrations were measured in *M. gulio* larvae following dietary exposure to different levels of E2, using either N feed or E feed, over treatment durations of 14 and 21 days (Figure 3). At 14 days, the testosterone levels in the control groups (0 mg/kg E2) were significantly different between the two feed types, with mean concentrations of 100.60 pg/mL (N feed) and 93.50 pg/mL (E feed), respectively (*p* < 0.05). At the 10 mg/kg hormone level, testosterone dropped significantly to 45.31 pg/mL in the N feed group and 9.65 pg/mL in the E feed group (*p* < 0.01). Similarly, at the 30 mg/kg level, concentrations were 23.34 pg/mL (N feed) and 16.16 pg/mL (E feed), with a statistically significant difference (*p* < 0.05). The most notable reduction was observed at 60 mg/kg, where testosterone levels declined to 12.83 pg/mL and 3.63 pg/mL for N and E feeds, respectively (*p* < 0.01).

At 21 days, testosterone levels continued to decline across all hormone doses. In the control groups (0 mg/kg E2), the mean testosterone concentrations were 90.18 pg/mL (N feed) and 92.31 pg/mL (E feed), showing no significant difference (*p* > 0.05). However, at 10 mg/kg, levels were 65.10 pg/mL in the N feed group and significantly lower at 10.86 pg/mL in the E feed group (*p* < 0.05). At 30 mg/kg, the values were 28.39 pg/mL and 18.58 pg/mL, respectively (*p* < 0.05). Finally, at the highest dose (60 mg/kg), testosterone levels were 27.90 pg/mL (N feed) and 3.63 pg/mL (E feed), again showing a highly significant difference (*p* < 0.01).

**Figure 4:**
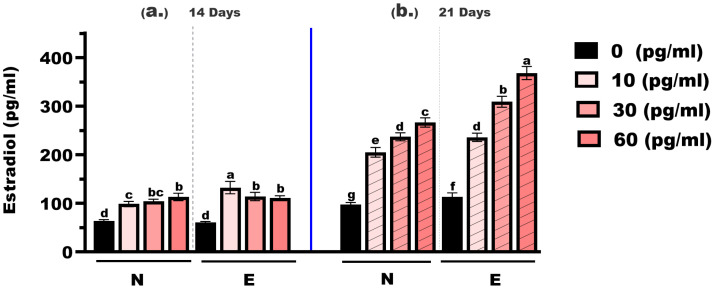
Estradiol concentrations in *Mystus gulio* larvae. Larvae were fed diets containing different 17β-estradiol levels (E2), delivered through two distinct feed types, conventional fishmeal-based feed (N) and hormonal complex-microencapsulated feed (E), over 14-day (**a**) and 21-day (**b**) treatment periods. Data are presented as mean ± standard error (SE). Concentrations are expressed in pg/mL. Data were analyzed using a one-way ANOVA, followed by Tukey’s multiple comparisons test. Different lowercase letters indicate statistically significant differences between groups (*p* < 0.05).

As shown in Figure 4, estradiol concentrations in *M. gulio* larvae varied significantly depending on hormone dose, feed type, and treatment duration. At the 14-day time point, estradiol levels in the control groups (0 mg/kg E2) were 63.98 pg/mL (N feed) and 60.42 pg/mL (E feed), with no statistically significant difference (*p* > 0.05). At 10 mg/kg, the levels increased to 98.65 pg/mL (N feed) and 132.60 pg/mL (E feed), showing a highly significant difference (*p* < 0.01). At 30 mg/kg, the concentrations were 104.30 pg/mL and 114.30 pg/mL, respectively, with no significant difference observed. Similarly, at 60 mg/kg, the values were 113.30 pg/mL and 111.20 pg/mL, again showing no significant difference between feed types.

At the 21-day mark, estradiol concentrations rose markedly across all treatment groups. In the 0 mg/kg E2 groups, the values were 97.84 pg/mL (N feed) and 113.20 pg/mL (E feed), with a statistically significant difference (*p* < 0.05). At 10 mg/kg, levels rose to 205.20 pg/mL and 236.10 pg/mL, respectively—also significantly different (*p* < 0.05). At 30 mg/kg, concentrations reached 237.10 pg/mL (N feed) and 309.30 pg/mL (E feed), showing a highly significant difference (*p* < 0.01). The highest concentrations were recorded at 60 mg/kg: 266.70 pg/mL (N feed) and 368.30 pg/mL (E feed), again with a highly significant difference (*p* < 0.01).

Across all hormone levels, estradiol concentrations increased with longer exposure time, and values recorded at 21 days were consistently higher than those at 14 days. Estradiol levels increased with both hormone concentration and exposure duration and were generally higher in the E feed groups, particularly after 21 days of feeding.

### 3.4. Gene Expression of Estrogen-Related Genes for Feminization of Mystus gulio Larvae

The relative expression of estrogen-related genes (*cyp19a*, *erb1*, and *erb2*) in *M. gulio* larvae was significantly influenced by both the concentration of E2 and the delivery method.

The relative expression of *cyp19a* exhibited a distinct dose-dependent upregulation in response to 17β-estradiol, with significant differences emerging between feed types as the treatment concentration increased. At 14 days, while low and medium doses (10 and 30 mg/kg) showed comparable expression levels between N feed and E feed diets (*p* > 0.05), a significant divergence occurred at the highest concentration. The E-60 group exhibited significantly higher *cyp19a* mRNA levels compared to the N-60 group (*p* < 0.001), indicating that microencapsulation enhanced hormone delivery efficiency at high doses early in the treatment period.

**Figure 5:**
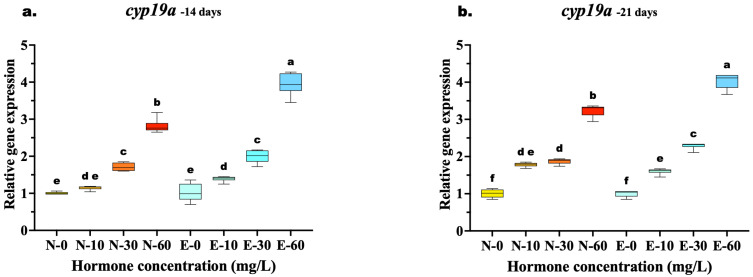
Relative gene expression of the *cyp19a* aromatase gene in *Mystus gulio* larvae. Larvae were fed diets containing different 17β-estradiol levels (E2), delivered through two distinct feed types, conventional fishmeal-based feed (N) and hormonal complex- microencapsulated feed (E), over 14-day (**a**) and 21-day (**b**) treatment periods. Data are presented as mean ± standard deviation (SD). Expression levels are relative to the control group (N-0 at each respective time point). Data were analyzed using a one-way ANOVA, followed by Tukey’s multiple comparisons test. Different lowercase letters indicate statistically significant differences within feed type and concentration level (*p* < 0.05).

By 21 days, the superiority of the E feed delivery system became even more pronounced across a wider range of concentrations. Unlike the 14-day results, the E-30 group significantly outperformed the N-30 group (*p* < 0.001), demonstrating that prolonged feeding allowed the E feed diet to achieve higher induction even at medium doses. The highest expression was consistently observed in the E-60 group, which remained significantly elevated compared to N-60 (*p* < 0.001). In contrast, the control groups (N-0 and E-0) showed no significant differences at any time point (Figure 5a,b).

The expression of *erb1* showed a significant upregulation in all hormone-treated groups compared to controls, though the comparative efficacy of the feed types varied with treatment duration. At 14 days, a clear dose–response relationship was evident, yet the highest concentration (60 mg/kg) resulted in similar expression levels for both feed types. Statistical analysis confirmed no significant difference between N-60 and E-60 (*p* = 0.986), suggesting that at this early stage and high dosage, receptor induction was maximized regardless of the delivery method. However, at the medium dose (30 mg/kg), the E feed group (E-30) was already significantly higher than the N feed group (N-30) (*p* < 0.001), indicating better efficiency at lower concentrations.

**Figure 6:**
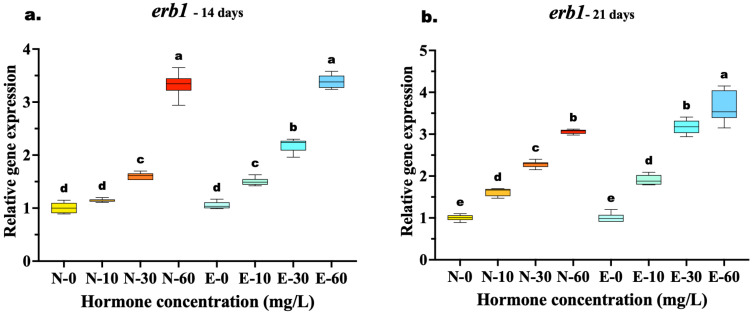
Relative gene expression of the *erb1* in *Mystus gulio* larvae. Larvae were fed diets containing different 17β-estradiol levels (E2), delivered through two distinct feed types, conventional fishmeal-based feed (N) and hormonal complex-microencapsulated feed (E), over 14-day (**a**) and 21-day (**b**) treatment periods. Data are presented as mean ± standard deviation (SD). Expression levels are relative to the control group (N-0 at each respective time point). Data were analyzed using a one-way ANOVA, followed by Tukey’s multiple comparisons test. Different lowercase letters indicate statistically significant differences within each feed type and concentration level (*p* < 0.05).

By 21 days, the advantages of microencapsulation became fully apparent at the highest dose. The E-60 group exhibited the highest overall expression, significantly surpassing the N-60 group (*p* < 0.001), which indicates that prolonged feeding with E feed diets sustains higher receptor activity than N feed. Furthermore, the efficiency of the E feed diet was highlighted by the fact that the medium dose E-30 induced expression levels statistically equivalent to those of the high dose of the N-60 (*p* = 0.934), suggesting that microencapsulation allows for effective feminization with lower hormone quantities (Figure 6a,b).

The transcriptional response of *erb2* mirrored the trends observed in *erb1*, with E feed demonstrating clear advantages in sustaining receptor upregulation. At 14 days, the highest concentration (60 mg/kg) yielded similar expression levels between the two feed types (N-60 vs. E-60, *p* = 0.067), suggesting a temporary saturation of the receptor response at early high doses. However, the superior bioavailability of the microencapsulated delivery was already evident at lower concentrations, where the E-30 group significantly outperformed the N-30 group (*p* < 0.001).

**Figure 7:**
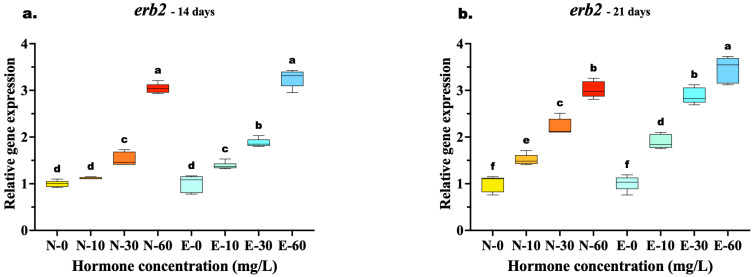
Relative gene expression of the *erb2* in *Mystus gulio* larvae. Larvae were fed diets containing different 17β-estradiol levels (E2), delivered through two distinct feed types, conventional fishmeal-based feed (N) and hormonal complex-microencapsulated feed (E), over 14-day (**a**) and 21-day (**b**) treatment periods. Data are presented as mean ± standard deviation (SD). Expression levels are relative to the control group (N-0 at each respective time point). Data were analyzed using a one-way ANOVA, followed by Tukey’s multiple comparisons test. Different lowercase letters indicate statistically significant differences within each feed type and concentration level (*p* < 0.05).

By 21 days, the divergence at the highest dose became statistically significant, with the E-60 group exhibiting the highest expression peak, surpassing the N-60 group (*p* = 0.002). Most notably, the E feed demonstrated superior potency: the expression level in the medium-dose group (E-30) was statistically equivalent to the high-dose N feed group (N-60) (*p* = 0.873) (Figure 7a,b).

## 4. Discussion

### 4.1. Preparation and Nutritional Evaluation of Hormonal Complex-Microencapsulated Feed for Mystus gulio Weaning Larvae

The successful preparation of hormonal complex-microencapsulated feed using a twin-screw extruder and double-layer conditioning system demonstrates the feasibility of delivering E2 in a stable and controlled manner during early larval development [23]. The resulting microparticles (250–300 µm) matched the ingestive capacity of *M. gulio* at the weaning stage, facilitating efficient feed uptake. Microencapsulation is well recognized for its ability to protect bioactive compounds such as hormones, vitamins, or probiotics from environmental degradation and to enhance their stability in aquatic systems [24].

Although direct quantification of hormone leaching into the water column was not performed, the efficiency of the delivery system is reflected in the bioaccumulation data. The encapsulation process not only protected the hormone from leaching but also allowed for uniform coating with oil–egg yolk emulsions to enhance palatability, an approach previously reported to improve feed acceptability and ingestion in fish larvae [25,26]. The significantly higher whole-body estradiol concentrations observed in the E feed group (Figure 4) provide functional evidence that the encapsulation matrix effectively minimized leaching. In N feed groups, the rapid disintegration of pellets often releases the steroid into the water before ingestion, reducing the effective dose. In contrast, the stability of the microencapsulated matrix ensured that a higher proportion of the bioactive compound reached the digestive tract, resulting in the elevated tissue concentrations observed. Nutritional analysis confirmed that the feed met standard larval requirements, including appropriate levels of protein, lipid, and essential amino acids, comparable to those reported for optimal larval development in other catfish species [27,28].

### 4.2. Evaluation of Amino Acid Profiles for Mystus gulio Weaning Larvae

The evaluation of amino acid profiles based on the A/E ratio and Ideal Protein Concept (IPC) models demonstrates that the experimental feed formulated for *M. gulio* larvae closely approximates the amino acid composition of natural food sources, such as fertilized eggs and fry [29]. The feed contained optimal levels of several essential amino acids, including lysine, leucine, and arginine, which are crucial for larval growth and development [30]. This approach allowed for the formulation of a diet that mimicked the species’ endogenous nutritional profile during early larval development.

Lysine, the most abundant essential amino acid in both fry and eggs, was well-represented in the experimental feed, accounting for approximately 14.6–16.7% of the total essential amino acids, matching the biological needs of croker (*Argyrosomus regius*) [31]. Leucine, an essential branched-chain amino acid, was also adequately present, supporting muscle protein synthesis during early development. The feed’s leucine content, which accounted for 4.10% of dry weight, was consistent with the natural leucine levels found in several fish fry and eggs [32]. Similarly, arginine, critical for immune function and protein synthesis [33], was present in the feed in an amount comparable to natural sources, further supporting the suitability of the feed for optimal larval development [34].

The amino acid composition of the formulated diet for *M. gulio* weaning larvae closely resembled that of natural references, including fry and fertilized eggs; however, certain essential amino acids were present at suboptimal levels. Methionine and cystine, both sulfur-containing amino acids, were notably lower than those found in natural sources. As these amino acids are essential for tissue synthesis, antioxidant defense, and methylation pathways, their deficiency may limit growth potential and stress tolerance [35]. This observation corresponds with previous reports identifying methionine as one of the most limiting amino acids in plant-based or semi-purified larval feeds [36,37]. Similarly, tryptophan, an amino acid vital for serotonin synthesis and stress regulation, was considerably lower in the formulated diet (0.43%) compared to fry (0.82%) and eggs (0.61%) [30,38]. Such deficiencies could impair the overall physiological resilience and immune performance of larvae under culture conditions. Therefore, while the experimental feed successfully reproduced the general amino acid profile of natural diets, further enrichment with methionine, cystine, and tryptophan would enhance its nutritional adequacy, supporting optimal growth, immune function, and survival of *M. gulio* during early weaning.

The amino acid composition of the formulated feed closely mirrored that of *M. gulio* fertilized eggs and fry, particularly in key growth-related amino acids such as lysine, leucine, and arginine [7,39]. Both the A/E ratio (amino acid to essential amino acid ratio) and the Ideal Protein Concept (IPC) are widely used to evaluate the nutritional quality of feeds and estimate amino acid requirements in aquaculture species [36,40]. The lysine content in the experimental feed, accounting for 14.6–16.7% of total essential amino acids, falls within the recommended range for optimal growth in freshwater fish larvae [41,42].

The relative abundance of leucine and arginine, as well as their IPC values compared to lysine, were also consistent with profiles reported in natural references such as fish eggs and the early-stage fry of related species [41,43]. These amino acids play critical roles in muscle protein synthesis and immune function, particularly during early development.

However, the formulated feed showed deficiencies in sulfur-containing amino acids, particularly methionine and cystine, which are essential for tissue development, antioxidant defense, and methylation pathways. This aligns with previous findings where methionine is frequently limited in plant-based or semi-purified diets for larvae unless supplemented.

### 4.3. Growth Performance, Feed Efficiency, and Feminization Success

The growth performance of *M. gulio* larvae in this study was significantly enhanced by the inclusion of E2, particularly at the highest tested concentration of 60 mg/kg. Both the conventional feed and the hormonal complex-microencapsulated feed supported high weight gain (WG), but the E feed version yielded slightly more consistent results in terms of specific growth rate (SGR) and feed efficiency (FE). These improvements align with previous studies reporting that low-to-moderate doses of estrogenic hormones can stimulate somatic growth in teleost fish, especially during early life stages [44]. In this study, SGR values exceeding 26%/day and FE values greater than 150% were recorded in the E feed 60 mg/kg group, highlighting the superior conversion of feed to biomass. Such values are indicative of efficient protein utilization and low metabolic loss, traits commonly observed when hormonal treatments are combined with optimized feed delivery systems [45]. The enhanced performance in the E feed groups is likely attributable to the controlled hormone release and reduced leaching associated with encapsulation, which ensures higher bioavailability of both nutrients and hormones [24,44].

The success of hormonal sex reversal relies heavily on administering the treatment during the labile period, the specific window of development when the undifferentiated gonad is responsive to exogenous steroids. In siluriform species, this window typically coincides with the onset of exogenous feeding [46]. In *M. gulio*, the gonads remain sexually undifferentiated at 3 dph. By initiating the E2 treatment at this stage, we ensured that the hormone was present prior to the molecular commitment to the testicular pathway. Our results confirm that the 21-day treatment duration effectively encompassed the critical period of morphological differentiation.

The high feminization rates (>98%) suggest that the treatment window from 3 to 24 dph fully covers the hormone-sensitive phase for this species. This is particularly relevant given that high hormone concentrations or prolonged exposure have been reported in other species to reduce survival or induce deformities [47]. The absence of such effects here may be due to the short-term exposure (14–21 days) and the protective effect of microencapsulation, which may mitigate hormonal stress. The feminization success achieved in this study was remarkable, with the highest female ratio (99.73 ± 0.04%) observed in the 60 mg/kg E2 group using E feed. This result is comparable or superior to feminization rates reported in other catfish species and freshwater finfish subjected to similar estradiol protocols. The comparable effectiveness of both feed types, 98.50% in the conventional group, demonstrates that the hormonal dose was effective, but microencapsulation further improved consistency, possibly due to enhanced hormone stability during digestion and absorption.

A relationship between hormone accumulation and phenotypic outcome was observed in the low-dose treatments, where the E feed 10 mg/kg group exhibited elevated somatic estradiol levels but failed to achieve significant feminization (approximately 53% female). This suggests that phenotypic sex reversal in *M. gulio* is governed by a threshold effect rather than a linear dose–response relationship.

Feminization outcomes showed a clear and highly effective sex reversal trend, particularly in groups receiving 60 mg/kg E2. The group fed E feed for 21 days reached a female ratio of 99.73 ± 0.04%, a remarkably high result indicating nearly complete feminization. Similarly, the group receiving the same hormone level with conventional feed achieved a female proportion of 98.50 ± 0.20%, which is statistically comparable. These findings demonstrate that both feed types can induce feminization effectively, but the hormonal complex-microencapsulated feed provides superior consistency and protection during delivery.

### 4.4. Gonadal Histology for Sex Differentiation

Gonadal morphology in *M. gulio* larvae, observed through histological sections, provided clear confirmation of phenotypic sex differentiation in response to E2 treatment (Figure 2). In E2-treated groups, especially at 60 mg/kg, ovarian development was prominent and characterized by the presence of cortical alveoli oocytes and early yolk granule stages. As evidenced by the histological analysis at the end of the experiment, which showed distinct ovarian cavity formation in the treated groups compared to the spermatogonia clusters in the untreated group, the histological integrity of the gonads serves as the definitive validation of sex reversal. The phenotypic females identified in the E2 treatment groups possess anatomically normal ovarian tissue rather than intersex or malformed gonads. These are well-established indicators of ovarian maturation in feminized teleost fish [48,49].

Additionally, the use of Davidson’s fixative and H&E staining proved effective for preserving gonadal tissue and visualizing key reproductive structures in early juveniles, a method commonly employed in sex differentiation studies in small-bodied fish species [50]. The ability to reliably distinguish male and female gonads using these tools strengthens the experimental assessment of feminization success and provides essential histological validation for the endocrine and performance-based results obtained in this study. Similar observations have been reported in various aquaculture species such as tilapia [51], catfish [52], and blotched snakehead [53], where hormone-induced feminization was confirmed via histological identification of ovarian tissue.

### 4.5. Expression of Estrogen-Related Genes and Their Role in Feminization

Overall, these results confirm that E2 is effective at promoting both growth and feminization in *M. gulio* larvae and that hormonal complex-microencapsulated feed offers practical advantages for improving feed performance, minimizing environmental leaching, and achieving near-complete monosex production, an essential strategy in modern aquaculture [54]. Hormonal and molecular analyses collectively demonstrate that E2 supplementation effectively modulates the endocrine system and activates the estrogen-signaling pathway responsible for feminization in fish larvae [44]. The gradual decline in testosterone levels with increasing hormone doses and prolonged exposure indicates a classical negative feedback effect on the hypothalamic–pituitary–gonadal (HPG) axis, leading to the suppression of androgen synthesis [55]. In contrast, estradiol levels increased in a dose- and time-dependent manner, particularly in larvae fed the hormonal complex-microencapsulated feed, confirming greater hormone bioavailability and retention through controlled release and reduced leaching [24,56]. The elevated estradiol concentrations corresponded closely with the upregulation of estrogen-related genes, especially *cyp19a*, *erb1*, and *erb2*, which play central roles in ovarian differentiation and estrogen responsiveness [57,58].

Research has demonstrated that estrogen exposure during early developmental stages enhances ovarian differentiation and suppresses testicular development, thereby increasing the proportion of females in the population [59]. For instance, in brown trout (*Salmo trutta*), oral administration of E2 has resulted in feminization rates of up to 86% [60]. While the feminization of fish using E2 has been well documented in several species, its application in scaleless fish [61], however, requires further study. Unlike scaled species, scaleless fish may absorb hormones more efficiently through their gills and intestinal walls, potentially leading to higher bioavailability and faster physiological responses. This physiological trait could enhance the efficacy of oral hormone treatments but could also increases the risk of adverse effects if dosing is not carefully managed [62]. As such, determining the appropriate dose and duration of E2 exposure in *M. gulio* is critical for ensuring successful sex reversal without compromising fish health or reproductive capacity. However, concerns remain regarding the long-term effects of exogenous estrogen exposure. Excessive hormone application may cause gonadal malformation, sterility, or reduced fertility in feminized fish [63,64]. Moreover, hormone residues released into the environment may disrupt wild fish populations, particularly in open-system aquaculture. These risks highlight the need for precise, controlled hormone delivery methods that maximize feminization success while minimizing environmental impact.

The enzyme aromatase, encoded by *cyp19a*, converts androgens to estrogens and thus serves as a key regulatory point in establishing female phenotypes [65]. Our results demonstrate that exogenous estradiol triggers a positive auto-regulatory loop, significantly upregulating *cyp19a* expression. Crucially, this upregulation was strictly dose- and delivery-dependent; significant induction occurred only when dietary inclusion reached a critical threshold (>30 mg/kg). This aligns with the “threshold hypothesis”, which posits that below a specific concentration, the molecular signal is insufficient to override the genetic male pathway [66], supporting enhanced aromatization activity and elevated estradiol synthesis under estrogen exposure [67]. For successful ovarian differentiation, exogenous estradiol must reach a critical concentration sufficient to trigger the permanent upregulation of the *cyp19a* gene [50]. The sustained high expression of *cyp19a* in the E-60 group at day 21, significantly higher than the N-60 group, suggests that microencapsulation prevents hormonal leaching, thereby maintaining the intracellular estrogen levels necessary to drive this permanent genetic shift.

The concurrent upregulation of *erb2* suggests receptor crosstalk and amplification of estrogenic signaling, as *erb2* is known to facilitate intracellular responses to elevated estrogen levels during gonadal differentiation [68]. This enhanced bioavailability is further corroborated by the expression profiles of estrogen receptors erb1 and erb2. Both receptors exhibited ligand-dependent activation, mediating the transcriptional response to the exogenous hormone [57]. A key finding of this study is the “dose-sparing” effect of microencapsulation: the expression levels of *erb1* and *erb2* in the E-30 were statistically equivalent to those in the high-dose N-60 by day 21. This indicates that the microencapsulated diet delivers the hormone so efficiently that a lower quantity is required to achieve maximal receptor saturation. By stabilizing the delivery of E2, microencapsulation ensures prolonged receptor activation (*erb1*/*erb2*), which, in turn, drives the continuous upregulation of *cyp19a*, ultimately leading to the near-total phenotypic feminization observed in the treatment groups.

Furthermore, while the small larval size precluded high-frequency blood sampling to generate a pharmacokinetic curve, the gene expression profiles offer molecular evidence of sustained hormone availability. The expression of estrogen receptor *erb2*, which is sensitive to circulating estrogen levels, was significantly higher in the microencapsulated treatment (Figure 6). This strong, persistent upregulation suggests that the microencapsulated matrix provided a continuous supply of the hormone to the target tissues, avoiding the rapid fluctuations often associated with uncoated dietary steroids [15,16]. Together, these gene expression patterns correspond to the observed hormonal changes, including reduced testosterone and increased estradiol, and confirm a coordinated molecular response that drives feminization.

## 5. Conclusions

The commercial aquaculture of *M. gulio* currently faces significant challenges related to sexual dimorphism, where mixed-sex culture results in inconsistent harvest sizes and suboptimal economic returns due to the slower growth of males compared to females. While hormonal feminization is a viable solution, traditional delivery methods such as feed top-dressing often suffer from leaching and inconsistent dosage, leading to variable success rates. This study addresses these critical industry problems by demonstrating that microencapsulated feed significantly enhances the delivery efficiency of 17β-estradiol. By preventing hormone leaching and ensuring precise dosage ingestion, the microencapsulation approach yielded superior growth rates and near-total feminization (99.73%) compared to conventional feeding methods. Consequently, this study establishes a reliable, resource-efficient protocol for producing monosex female populations, which is essential for scaling up *M. gulio* production and improving farm profitability.

Future research should focus on assessing the reproductive performance of these neo-females in the F1 generation to ensure long-term viability. Additionally, further investigation is required to monitor potential hormone residues in culture water and sediments, ensuring that this intensive production method remains environmentally sustainable. Although this study indirectly suggests reduced environmental contamination through improved hormone uptake and biological efficiency, future studies should directly quantify hormone residues in the rearing water to validate the environmental safety of this microencapsulation technique.

## Figures and Tables

**Table 1:** DNA primer for RT–qPCR and amplicon sizes.

Gene		Primer Sequence (5′–3′)	GenBank (Reference Sequence)	Amplicon Size (bp)
*cyp19a*	For	CCTCGTCGTTACTTCCAGCC	KX787079	192
	Rev	TCAAACCCTTATGGAGGCAAA		
*erb1*	For	AACTGGTTTTCCCAGACACAC	KT832704	290
	Rev	GCGTATAATGCCAACACCCTC		
*erb2*	For	CGCCAACACTACTCACAGCA	KT832703	298
	Rev	AAAGATTTTGGGAGCGGGAG		
*β-actin*	For	AAGTACCCCATTGAGCACGG	XM_027148463	464
	Rev	TCAAGGGCAACGTAGCAGAG		

**Table 2:** Chemical composition (g/100 g dry matter or %) of *M. gulio* fry, fertilized eggs, and the experimental weaning feed.

Chemical Composition	Fry (Fresh Frozen)	Fertilized Egg (Fresh Frozen)	Feed
Dry matter	20.62	44.62	90.36
Total crude protein	65.83	57.23	39.38
Total crude fat	1.66	5.34	2.14
Essential amino acids			
Arginine	3.65	5.14	3.59
Histidine	1.42	2.03	1.42
Isoleucine	2.12	3.42	2.39
Luecine	3.95	5.87	4.1
Lysine	4.65	5.23	3.66
Methionine	1.21	2.01	1.4
Cystine	0.53	1.15	0.8
Phenylalanine	2.28	2.64	1.85
Tyrosine	2.11	2.05	1.43
Threonine	2.64	2.94	2.05
Tryptophan	0.82	0.61	0.43
Valine	2.48	2.84	1.98
Total essential amino acids	27.86	35.93	25.1
Non-essential amino acids			
Alanine	2.84	4.36	3.05
Aspartic	5.2	5.82	4.07
Glutamic	5.14	8.83	5.47
Glycine	1.67	2.21	1.47
Proline	1.81	2.55	1.78
Serine	2.91	5.61	3.92
Total non-essential amino acids	19.57	29.38	19.76
Total amino acid	47.43	65.31	44.86

**Table 3:** Probability (*p* values) and F-ratios for the main and interaction effects of 17β-estradiol (E2) hormone dose (HD), feed type (FT), and hormone treatment period (TP) on growth performance parameters (WG, SGR, FE, and sex ratio) of *Mystus gulio* fry reared in fiber tanks for 28 days, analyzed using three-way ANOVA.

Factor	Source	Degrees of Freedom	F Ratio	*p* Value	Factor	Source	Degrees of Freedom	F Ratio	*p* Value
Survival	HD	3	7.084	*p* < 0.001	SGR	HD	3	30.84	*p* < 0.001
	FT	1	0.038	*p* = 0.958		FT	1	36.23	*p* < 0.001
	TP	1	0.003	*p* = 0.846		TP	1	76.26	*p* < 0.001
	HD×FT	3	0.313	*p* = 0.816		HD×FT	3	15.14	*p* < 0.001
	HD×TP	3	0.202	*p* = 0.894		HD×TP	3	8.632	*p* < 0.001
	FT×TP	1	0.015	*p* = 0.904		FT×TP	1	1.305	*p* = 0.262
	HD×FT×TP	3	0.258	*p* = 0.855		HD×FT×TP	3	29.61	*p* < 0.001
WG	HD	3	29.94	*p* < 0.001	FE	HD	3	33.34	*p* < 0.001
	FT	1	6.482	*p* < 0.001		FT	1	51.34	*p* < 0.001
	TP	1	81.56	*p* = 0.011		TP	1	242.6	*p* < 0.001
	HD×FT	3	3.07	*p* = 0.028		HD×FT	3	6.309	*p* = 0.002
	HD×TP	3	7.046	*p* < 0.001		HD×TP	3	1.972	*p* = 0.138
	FT×TP	1	7.027	*p* = 0.008		FT×TP	1	1.025	*p* = 0.319
	HD×FT×TP	3	81.41	*p* < 0.001		HD×FT×TP	3	29.41	*p* < 0.001
Sex ratio	HD	3	50.16	*p* < 0.001					
	FT	1	9.673	*p* = 0.004					
	TP	1	0.882	*p* = 0.355					
	HD×FT	3	0.008	*p* = 0.999					
	HD×TP	3	3.313	*p* = 0.032					
	FT×TP	1	0.11	*p* = 0.742					
	HD×FT×TP	3	0.459	*p* = 0.713					

Abbreviations: HD = hormone dose (mg/kg); FT = feed type (N = conventional feed; E = hormonal complex-microencapsulated feed); TP = treatment period (days); WG = weight gain (g); SGR = specific growth rate (%/day); FE = feed efficiency (%); Sex ratio = percentage of phenotypic females.

## Data Availability

The original contributions presented in this study are included in the article/Supplementary Material. Further inquiries can be directed to the corresponding author.

## Supplementary Materials

The following supporting information can be downloaded at: https://www.mdpi.com/article/10.3390/ani16020268/s1, Figure S1: Measurement and microscopic visualization of feed particle size and morphology. (a) Conventional feed (N): Bulk feed pellet on a 15-mL petri dish (left) and particle morphology under a light compound microscope (10× magnification) (right). (b) Hormonal Complex-Microencapsulated Feed (E): Bulk feed pellet on a 15-mL petri dish (left) and particle morphology under a light compound microscope (10× magnification) (right).

## Abbreviations

The following abbreviations are used in this manuscript:

E2	17β-estradiol
E	hormonal complex-microencapsulated feed
N	normal feed, conventional fishmeal-based feed
dph	days post hatch
H&E	hematoxylin and eosin
*cyp19a*	Aromatase gene
*erb1*	estrogen receptor β1
*e**rb2*	estrogen receptor β2
