# Supplementary material for: The Effects of Hormone Diets with Different 17β-Estradiol Levels on Growth and Feminization in Long-Whiskered Catfish (Mystus gulio) Larvae Using Conventional and Microencapsulated Feed
Source: Animals (Basel). 2026 Jan 15;16(2):268. doi: 10.3390/ani16020268 (PMC12837676; doi:10.3390/ani16020268)
Supplement: Supplementary file 1 [file animals-16-00268-s001.zip › animals-4011529-supplementary.pdf]

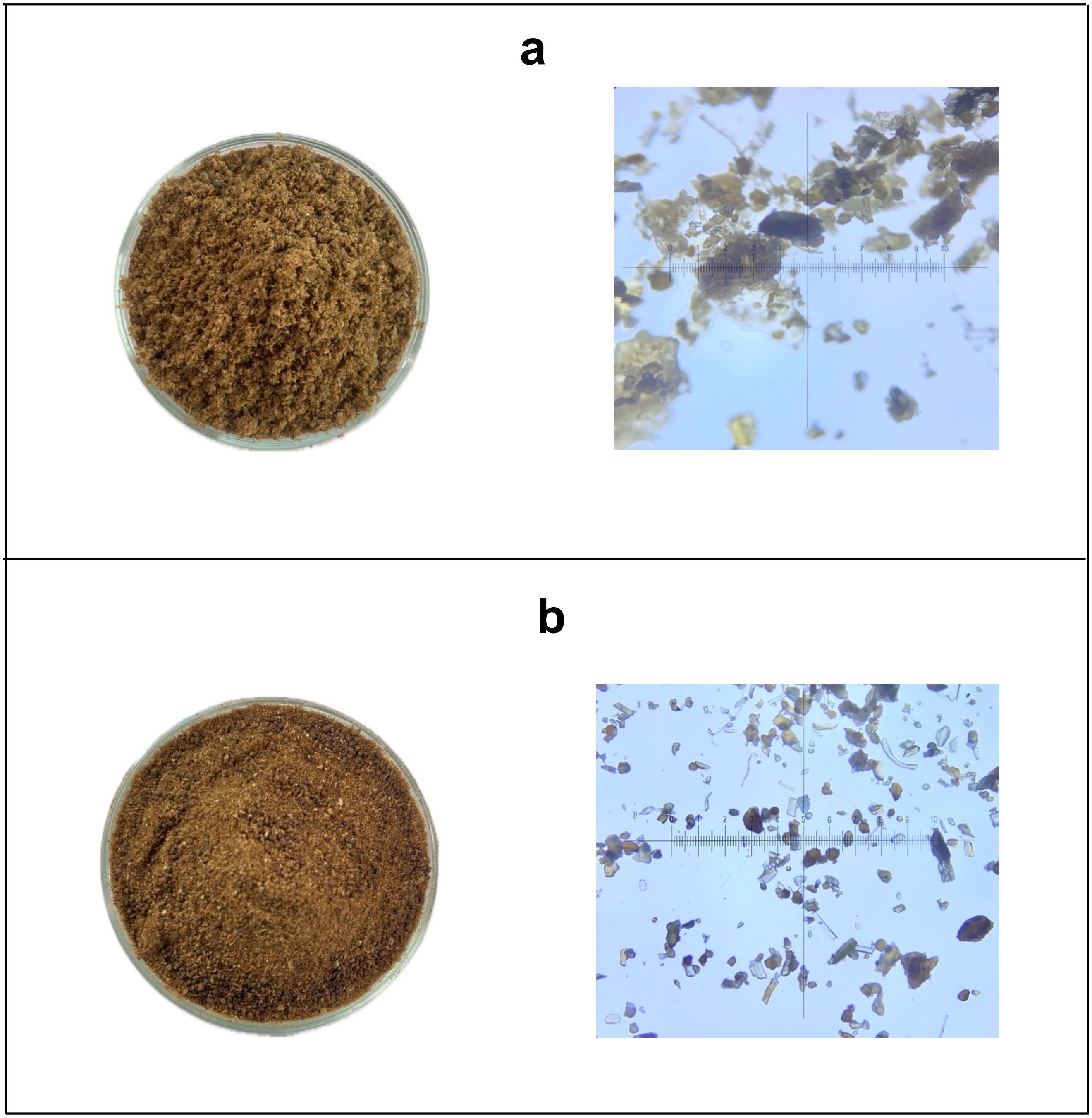

Figure S1. Measurement and microscopic visualization of feed particle size and morphology. (a) Conventional feed (N): Bulk feed pellet on a 15-mL petri dish (left) and particle morphology under a light compound microscope (10× magnification) (right). (b) Hormonal Complex-Microencapsulated Feed (E): Bulk feed pellet on a 15-mL petri dish (left) and particle morphology under a light compound microscope (10x magnification) (right).
